# Delayed gratification in New Caledonian crows and young children: influence of reward type and visibility

**DOI:** 10.1007/s10071-019-01317-7

**Published:** 2019-10-19

**Authors:** Rachael Miller, Anna Frohnwieser, Martina Schiestl, Dakota E. McCoy, Russell D. Gray, Alex H. Taylor, Nicola S. Clayton

**Affiliations:** 1 Department of Psychology, University of Cambridge, Cambridge, UK; 2 School of Psychology, Auckland University, Auckland, New Zealand; 3 Max Planck Institute for the Science of Human History, Max Planck Society, Jena, Germany; 4 Department of Organismic and Evolutionary Biology, Harvard University, Cambridge, USA

**Keywords:** Delayed gratification, Corvids, Children, Self-control

## Abstract

Self-control underlies cognitive abilities such as decision making and future planning. Delay of gratification is a measure of self-control and involves obtaining a more valuable outcome in the future by tolerating a delay or investing a greater effort in the present. Contextual issues, such as reward visibility and type, may influence delayed gratification performance, although there has been limited comparative investigation between humans and other animals, particularly non-primate species. Here, we adapted an automated ‘rotating tray’ paradigm used previously with capuchin monkeys to test for delay of gratification ability that requires little pre-test training, where the subject must forgo an immediate, less preferred reward for a delayed, more preferred one. We tested New Caledonian crows and 3–5-year-old human children. We manipulated reward types to differ in quality or quantity (Experiments 1 and 2) as well as visibility (Experiment 2). In Experiments 1 and 2, both species performed better when the rewards varied in quality as opposed to quantity, though performed above chance in both conditions. In Experiment 1, both crows and children were able to delay gratification when both rewards were visible. In Experiment 2, 5-year-old children outperformed 3- and 4-year olds, though overall children still performed well, while the crows struggled when reward visibility was manipulated, a result which may relate to difficulties in tracking the experimenters’ hands during baiting. We discuss these findings in relation to the role of contextual issues on self-control when making species comparisons and investigating the mechanisms of self-control.

**Electronic supplementary material:**

The online version of this article (10.1007/s10071-019-01317-7) contains supplementary material, which is available to authorized users.

## Introduction

Self-control is critical for humans and other animals, as it underlies effective decision-making and future planning, and ensures that individuals achieve goal-directed behaviour (Diamond 2013; McCormack and Atance 2011; Santos and Rosati 2015). One aspect of self-control is the ability to delay gratification and involves obtaining a more valuable choice over a less valuable one, by tolerating a delay or investing greater effort (or both) to obtain the more valuable outcome (Beran et al. 2016b). In children, delay of gratification is influenced by development, in that it improves between ages 3 and 5 years (Hughes 1998; Zelazo 2003). It shows high individual variation and correlates with some measures of success in later life, like social and academic competence (Mischel et al. 1989), though see a recent critique of previous findings using the standard ‘marshmallow’ task: (Watts et al. 2018). Intelligent decision-making is also important for other animals in a variety of social and foraging contexts, including inhibiting approaching food or mates in the presence of a competitor, or during tool use. Previous non-human studies have indicated large diversity of responses within the same species, see a recent review of delay of gratification in corvids (members of the crow family), parrots and non-human primates in Miller et al. (2019), so self-control does not appear to be shared equally between all species or individuals. In children (Mischel et al. 1972, 1989) and chimpanzees (*Pan troglodytes*: Beran and Hopkins 2018), delay of gratification links to measures of general intelligence.

Delay of gratification has been investigated in human and non-human species using various paradigms, for example, the exchange and accumulation paradigms (Beran 2018; Dufour et al. 2007, 2012; Parrish et al. 2014; Pelé et al. 2011; Steelandt et al. 2012). In these tasks, subjects are required to choose between two reward options, one available immediately and one following a delay (delay choice tasks) or required to sustain the decision to delay gratification while the immediate reward is present or already possessed during the delay (delay maintenance tasks) (Ainslie 1974; Beran and Evans 2006; Tobin et al. 1993, 1996). Subjects may be required to tolerate delays from seconds to minutes (Miller et al. 2019). In the exchange paradigm, subjects may be required to swap rewards or tokens with an experimenter or conspecific to acquire the ‘better’ reward (Beran et al. 1999). In the accumulation paradigm, rewards accumulate at a steady rate within the subject’s reach until they touch or consume them (Beran et al. 2016a). These and other delayed gratification paradigms have been used to test self-control in a number of species, including non-human primates, corvids and parrots, typically in single-species studies. For further details of traditional delayed gratification paradigms and species tested, see Beran (2018) and Miller et al. (2019) for recent reviews.

Performance in delayed gratification tasks may be influenced by contextual or methodological issues. Examples of contextual issues include reward type (e.g. reward quality and quantity) and reward visibility (e.g. immediate or delayed reward not visible). Both aspects are likely to be ecologically relevant for humans and non-human species. For example, delayed options in everyday life may be more abstract than immediate ones, and require memory representation rather than direct perceptual contact (Perdue et al. 2015). This can be observed in tool using species, who show the ability to delay gratification using tools to access out of reach food items instead of only eating immediately available food. Few studies have tested the influence of reward quality and quantity on performance—those that have are primarily in corvids and parrot species. Specifically, using the exchange and accumulation paradigms, carrion crows (*Corvus corone*), common ravens (*Corvus corax*), Goffin’s cockatoos (*Cacatua goffiniana*), kea (*Nestor notabilis)*, and African grey parrots (*Psittacus erithacus*) performed similarly as some non-human primates when the delayed reward was of higher quality than the immediate reward (Auersperg et al. 2013; Dufour et al. 2007, 2012; Hillemann et al. 2014). However, like chimpanzees (Beran et al. 2016b) and capuchin monkeys (*Sapajus apella*) (De Petrillo et al. 2015), these corvid and psittacine (members of the parrot order) species appear to struggle with delay of gratification tasks when the reward is of higher quantity (Auersperg et al. 2013; Dufour et al. 2007, 2012; Hillemann et al. 2014). These findings may be related to issues with quantity discrimination or to practical constraints of caching or transporting large quantities of food, which may encourage competition from others.

Reward visibility influences delay of gratification in humans, particularly in children, but also in great apes (Beran and Evans 2006; Kochanska et al. 2001; Mischel et al. 1972; Vlamings et al. 2006). In children, when the immediate reward is visible (and delayed reward not visible), performance typically suffers, whereas when the delayed reward is visible (and immediate reward is not), this sometimes improves self-control (Mischel et al. 1972). However, in capuchin monkeys, subjects continued to perform well even when the delayed option was not visible (Perdue et al. 2015).

The influence of contextual and methodological issues on performance has primarily focused on comparisons in primate species. Monkey performance on delay of gratification tasks varies across studies and paradigms, as well as in comparison with other primate species, like apes, potentially due to contextual and methodological issues (Addessi et al. 2011; Paglieri et al. 2013). To shed light on this area, Bramlett et al. (2012) introduced a novel task using a rotating tray that successively presented rewards of varying quality and quantity within reach, which, unlike most other paradigms, such as the exchange paradigm that requires subjects to learn to exchange rewards or tokens with an experimenter or conspecific, requires little pre-test training. They found that capuchins were able to let the first reward go past and wait for the second one, if it was a better or bigger reward. These findings were consistent across various short delay lengths and reward magnitudes (Bramlett et al. 2012). When reward visibility (though not reward type) was manipulated in a further study, capuchins still performed well, even when only the immediate, less preferred option was visible and the delayed option was hidden, or the baiting process took place out of sight of subjects (Perdue et al. 2015). The authors highlight that temporal delays are intuitive in this design as the subjects can directly observe the rewards moving closer to them (Perdue et al. 2015). They appeared more likely to understand the nature of the task and be able to make an informed decision about whether or not to take the immediate reward. Therefore, this automated rotating tray paradigm may be useful for making further comparisons with other species, as it reduces task demands by removing the added complexity involved in some of the other delayed gratification paradigms, such as extensive training and/or testing, exchanging with a human for non-human subjects or tracking an accumulation of rewards.

Few studies have directly compared delayed gratification performance of humans and non-human species using similar paradigms. One study that compared the performance of adult humans with chimpanzees and bonobos (*Pan paniscus*) indicated that humans are less tolerant of delays than great apes in waiting for food (Rosati et al. 2007). We note that some researchers have expressed concerns with using reaches toward food in such tests with regard to potential influences on species comparisons (Beran 2018). Another example is the exchange paradigm tested in separate studies with children (Steelandt et al. 2012), chimpanzees (Dufour et al. 2007), as well as common ravens and carrion crows (Dufour et al. 2012), indicating ability to delay gratification of varying degrees in these species. Specifically, corvids were able to tolerate delays of up to 5 min (Dufour et al. 2012) and chimpanzees up to 4 min for rewards of 2, 4 or 8 times the size of the initial food item (Dufour et al. 2007). Chimpanzee waiting time increased to 8 min if the reward was 40 times larger than the initial item (Dufour et al. 2007). In testing 2–4-year-old children, older children were able to tolerate longer delays than younger ones, though children as young as 2 years could wait up to 16 min (Steelandt et al. 2012). However, we note that these studies did not explore the role of contextual issues on performance, nor directly compared performance across the species.

Here, we adapted the rotating tray paradigm introduced by Bramlett et al. (2012) to test delay of gratification in New Caledonian crows (*Corvus moneduloides*) and 3–5-year-old human children—the latter age range selected as previous child development studies indicate that self-control typically develops between 3 and 5 years (Mischel et al. 1989). Corvids shared a common ancestor with primates over 300 million years ago, but show comparable performance with primates in some cognitive tasks, including in some cases, with young children (Cheke et al. 2012; Emery and Clayton 2004). New Caledonian crows routinely use tools in both foraging and non-foraging contexts, and have performed impressively across a wide range of problem-solving tasks (Gruber et al. 2019; Hunt 1996; Jelbert et al. 2015, 2019; Taylor et al. 2012; von Bayern et al. 2009). We assessed performance in crows and children using this automated paradigm. Specifically, in the first experiment, we examined the influence on performance of rewards varying in quality and quantity, and, in the second experiment, we manipulated the visibility of the reward.

We tested both species using the same apparatus and setup, though note that there were differences in methodology between species as outlined in the “Materials and methods” and “Discussion” sections, which may have affected our results and interpretation (Leavens et al. 2017). For example, we used food rewards for crows and stickers for children, as we were not allowed to give children food, and sessions were structured differently for children and crows due to the availability of both species, i.e. we had access to many children for short periods of time, as schools generally do not allow children to be taken out of class for more than half an hour per day, but a much smaller sample size of crows over a longer period of time. These time restrictions are also the main reason for differences in training, as children could only receive a very short training session aided by verbal instructions, while crows had to be trained non-verbally over the course of multiple days, which also lead to crows being more familiar with the setup by the time testing commenced. This study, therefore, explores each species’ ability to delay gratification in the rotating tray paradigm and tests this ability in relation to reward type and visibility. With regard to reward type, we predicted that crows and children would perform better when rewards differed in quality than quantity, following previous studies with other corvid species, chimpanzees and capuchins (Beran et al. 2016b; Bugnyar et al. 2012; De Petrillo et al. 2015; Dufour et al. 2012; Hillemann et al. 2014). With reward visibility, we expected that reward visibility would influence performance in children and crows, as reflected by previous child and most other primate research, however, we did not predict the direction of this influence, given that different primate species show different results when it comes to reward visibility, with some species performing better when only the delayed reward is visible and others performing better when only the immediate reward is visible (Beran and Evans 2006; Kochanska et al. 2001; Perdue et al. 2015). Additionally, we expected to find developmental differences in performance in children, following a similar trajectory as other delay of gratification paradigms (Mischel et al. 1989; Steelandt et al. 2012).

## Materials and methods

### Subjects

The bird subjects were nine New Caledonian crows caught from the wild (at location 21.67° S 165.68° E) on Grand Terre, New Caledonia, for temporary holding in captivity on the Island for non-invasive behavioural research purposes from April to July 2018. There were five males and four females, based on sexual size dimorphism (Kenward et al. 2004), of which four were adults and five were juveniles (less than 1 year old) (Online Resource 1). The birds were housed in a 10-compartment outside aviary, with compartments differing in size, though all at least 2 × 3 × 3 m, containing a range of natural enrichment materials, such as logs, branches and pinecones. Subjects were tested individually in temporary visual isolation from the group. The birds were generally not food deprived and their daily diet consisted of meat, dog food, eggs and fruit, with water available ad libitum. The birds maintained at or above capture weights during their stay in captivity. The birds were acclimatised to the aviaries in April and habituated to the experimental apparatus in May 2018. All birds completed the full study in June–July 2018. All birds took part in several other experiments during their stay in captivity, including making forced two-choices (e.g. between two tools or food types) and interacting with artificial apparatuses (e.g. Gruber et al. 2019). At the end of their research participation, birds were released at their capture sites. A previous study indicated that New Caledonian crows housed temporarily in a similar situation as the present study successfully reintegrated into the wild after release (Hunt 2016).

In addition, there were 61 child subjects aged between 3 and 5 years: 20 3-year olds (mean: 3.65 years; range: 3.01–3.98 years), 21 4-year olds (M: 4.68 years; R: 4.05–4.99 years) and 20 5-year olds (M: 5.34 years; R: 5.05–5.87 years), of which 31 were male and 30 were female. Children were recruited and tested at seven preschools and primary schools in Cambridgeshire and Buckinghamshire, serving predominantly white, middle-class communities, between March and June 2018. All children tested completed the full study; they were tested in temporary visual isolation from other children. For some of the younger children, a member of staff was present in the room, but did not interact with the child.

### Ethics statement

All procedures performed in studies involving human participants were in accordance with the ethical standards of the European Research Council Executive Agency Ethics Team (application: 339993-CAUSCOG-ERR) and University of Cambridge Psychology Research Ethics Committee (pre. 2013.109), and with the 1964 Helsinki Declaration and its later amendments or comparable ethical standards. Informed written consent was obtained from legal guardians prior to participation of the child. The parents of the children identified in the online resources movie gave their informed written consent for this information to be published. For the animal research, all applicable international, national and/or institutional guidelines for the care and use of animals were followed. The New Caledonian crow research was conducted under approval from the University of Auckland Animal Ethics Committee (reference number 001823) and from the Province Sud with permission to work on Grande Terre, New Caledonia, and to capture and release crows. Whoosh nets were used to catch all birds on private land with landowner permission and were released at the capture sites at the end of testing.

### Data availability

The full dataset is available on Figshare: https://figshare.com/s/2c0c48488b4ea58adfa2.

### Additional information

The authors declare no competing interests.

### Apparatus

**Fig. 1:**
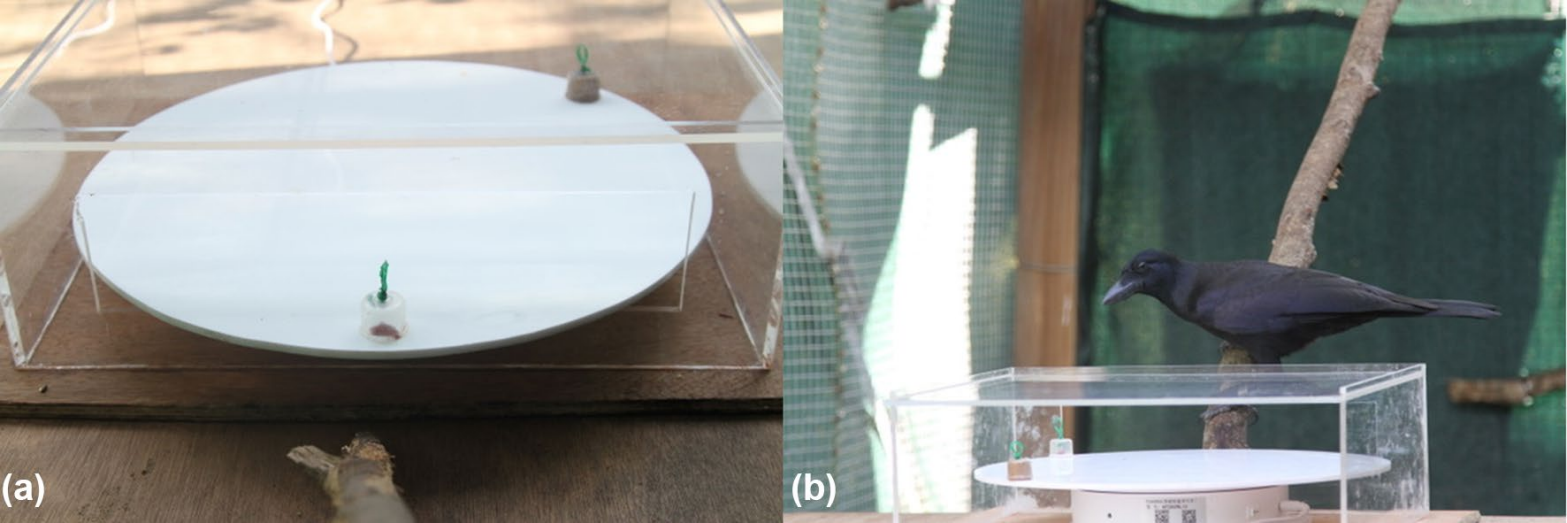
**a** Rotating tray with two containers (one transparent, one opaque), **b** crow subject approaching apparatus with containers in their starting positions (location 1, 5 s delay, and location 2, 15 s delay)

We used a 38-cm diameter elevated revolving disk, mounted on top of a rotation device moving at a speed of 68 s per revolution operated with a remote control (Fig. 1). The revolving disk was contained within a transparent Perspex box (41 cm × 34 cm × 14 cm) with a 29 cm × 7 cm rectangular opening at the front, to prevent subjects from taking rewards before they were located directly in front of the subject. Two small plastic containers holding the rewards were positioned so that the first container at location 1 would reach the subject after 5 s, and the second container at location 2 after 15 s. These delays were selected to be comparable to the previous monkey studies using a similar paradigm (Bramlett et al. 2012; Perdue et al. 2015). For the crows, the rewards were placed directly onto the rotating tray to ensure ease of access and visibility. Both locations were always baited simultaneously. For each trial, an experimenter entered the aviary in which the experiment took place to bait the apparatus and leave a very small food reward on a perch in front of it. Then, the experimenter left the aviary and closed the door, so they could not be seen by the subject. The rotating tray was started via remote control as soon as the bird sat on the perch in front of the apparatus and consumed the food reward, assuring that all birds were in the same starting position for each trial. The rotation was stopped by the experimenter when the subject took the reward from location 1 or 2—they were, therefore, only allowed to make one choice. Birds usually came to the perch within a few seconds after the experimenter left the aviary, and trials were discontinued if the bird refused to come for several minutes.

In the quality condition, the most preferred item for the crows was a small piece of meat and for the children a large glittery picture sticker, while the least preferred item for the crows was a small piece of apple and for the children a white, square sticker. For the quantity condition, the larger quantity for the crows was a piece of meat approx. 4 × the size of the smaller piece of meat used for the smaller quantity. For the children, the larger quantity was four mini-picture stickers with one mini-picture sticker for the smaller quantity. For crows, small pieces of meat were used during training. For the children, we also used an ‘ok’ reward for training, which was a yellow smiley sticker, to reserve the most and least preferred stickers for testing only and so maintain motivation during testing (as all trials were conducted in one session).

### Preference test

We first checked the preferences for the most and least preferred (quality) and large and small amount (quantity). We presented the choice of most vs. least quality, and large vs. small quantity, by placing both options on the table and allowing the subject to pick one option. For the crows, we ran sessions of 10 trials per session, until the subject selected correctly on 17 of 20 trials across two sessions, i.e. selected the better quality and larger quantity, up to a maximum of 10 sessions per condition, which all crows finished in 2 sessions (20 trials). The crows were allowed to only select one option on every trial by the experimenter opening the door to the aviary and stepping inside as soon as the first choice was made, which caused the crows to leave the table where the rewards were presented. For the children, we ran one trial per condition, as we have found in pilot testing and in previous studies (unpublished) that the majority of children showed clear preferences for the ‘better’ option in a single choice, and to avoid them losing interest in the rewards. In a previous study, we also found that children consistently selected the least preferred sticker in the absence of the most preferred sticker (e.g. over a piece of tissue). Due to these methodological differences, crows had more pre-test experience with the reward types used in the study.

### Experiment 1: the influence of reward type

#### Training

For both children and crows, we ran forced trials, where a reward was placed at one location only—location 1 or 2, with no reward at the other location within each trial. This training step assured that the subjects were able to retrieve the reward from the rotating disk, and paid attention when rewards became accessible. For the children, this was two trials—one per location. For the crows, sessions of 10 trials were run until the subject successfully retrieved the reward in 80% of trials (18/20 trials) across 2 sessions. Each crow received a maximum of 10 sessions (100 trials) and were discounted from further testing if they failed to reach criterion at this stage. Note that all crows passed the training criteria within 20–50 trials (Online Resource 2). For the children, we also first ran a demonstration trial, where the experimenter started the tray rotating and asked the child to select the container when it arrived in front of them in their reach, and explained that they could select only one container and that the tray would rotate only once.

#### Testing

In test trials, the better/bigger reward was at location 2 and the poorer/smaller reward was at location 1, while in control trials this was reversed: the better/bigger reward was at location 1 and the poorer/smaller reward at location 2. Tests were run for each condition (quality/quantity), with half of the subjects receiving all test sessions in the quality condition before being tested in the quantity condition, and vice versa for the other half. Both rewards were visible. For the crows, a total of 60 trials were run, 30 for each condition, made up of 3 sessions that included 3 control and seven test trials. Therefore, 9 control trials and 21 test trials were run for each condition. Control trials were randomly interspersed across sessions. To pass each condition, the crow subject was required to make the correct choice, i.e. select the delayed reward, in 16 of 21 test trials (significant with two-tailed exact Binomial test). Each bird received up to three test sessions per day, depending on time constraints and motivation. For the children, due to time and access restraints, we ran eight trials in total: two test trials and two control trials per condition, to be able to explore performance on a group level (e.g. comparing age groups).

### Experiment 2: the influence of reward visibility

#### Training

In Experiment 2, the same setup and apparatus was used as in Experiment 1. For the crows, we ran two training steps with forced trials. In training 1, a reward was placed in one location (1 or 2) and small transparent caps were placed on both locations, covering the reward. This was done to ensure that the crows were able to lift the caps and paid attention to the reward underneath them. The subject was required to lift the cap to obtain the reward. In training 2, to ensure that the subjects understood that rewards could be underneath either opaque or transparent caps and could remember the location of the hidden reward, a reward was placed in one location (1 or 2) with a transparent cap on one location and an opaque cap on the other location. As in Experiment 1 training, we continued with training until the subject selected correctly in 18 of 20 trials over two consecutive sessions when both caps were transparent and when one cap was clear and one was opaque, to a maximum of 100 trials. If they failed to do so within 100 trials, they were discounted from testing.

For the children, we ran four forced trials with a reward placed at one location in one opaque container, with no reward in the second location in the other opaque container to check that they could remember the location of a hidden reward. Both containers were touched simultaneously while baiting and then the opaque lids were closed so that the reward was not visible once baiting was complete and the rotation started. We used the medium reward placed at location 1 in two trials, and location 2 in two trials. If the child failed to correctly locate the hidden reward in any of the first four trials, then two additional trials (one trial at location 1, one at location 2) were run.

The crow training differed from the child training for Experiment 2, as the crows struggled considerably with locating the hidden reward when both containers were opaque, though were able to do so when one container was opaque and the other transparent. The crows were wild-caught for temporary holding in captivity, and although were acclimatised and comfortable moving around the aviary and coming to the apparatus when the experimenter was outside the aviary, they would not remain at the table directly in front of the apparatus while the experimenter was present baiting it. Therefore, the view of baiting and the delay between observing baiting and making their choice was different for the crows than the children. The children would sit in front of the apparatus during baiting, while the crows always had the possibility to observe baiting though would usually perch on a branch approx. 3 m from the apparatus table. Crows were generally afraid of humans approaching them closely and, therefore, did not observe baiting in the majority of trials. However, as they had previously participated in daily training and other studies, they were comfortable approaching different setups on the experimental table and interacting with apparatuses when no experimenter was present in the compartment. A previous study (Jelbert et al. 2016) has shown that crows can learn to observe baiting through extensive training when the experimenter was hidden behind a screen and only their hands were visible, which was not the case in this experiment, as the experimenters had to enter the aviary to bait the apparatus.

#### Testing

**Fig. 2:**
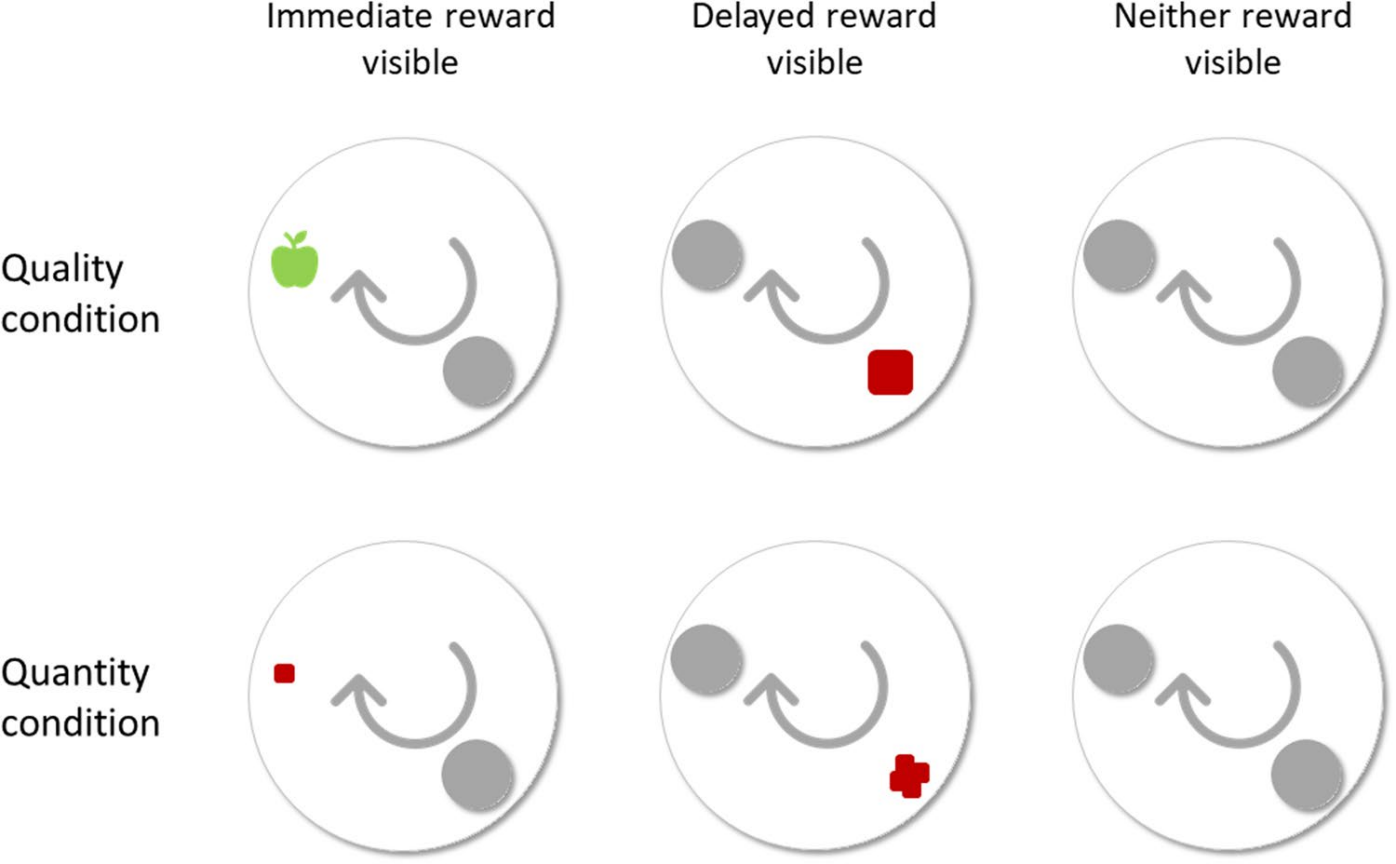
Testing types for Experiment 2

We ran test trials, with the better/bigger reward in location 2 and poorer/smaller reward at location 1, and control trials, with the better/bigger reward in location 1 and poorer/smaller reward at location 2, for both conditions (quality/quantity). A reward was placed in both locations in view of the subject. In test step 1, the container in location 1 had a transparent lid, while the container in location 2 had an opaque lid (Fig. 2). Therefore, only the immediate reward was visible when the rotation started. In test step 2, the container in location 1 had an opaque lid, and the location 2 had a transparent lid, so only the delayed reward was visible when the rotation started. In test step 3, both containers had an opaque lid, therefore, neither reward was visible when the rotation started. Test step 3 was only run with children, not crows, due to the aforementioned difficulties of crows observing the experimenter during baiting. Half of children received step 1, then step 2 and then step 3, the other half received step 2, step 1 and then step 3. Half of children received all quality trials first; the other half received all quantity trials first.

For the crows, subjects received a total of 30 trials across 3 sessions per condition. Each session consisted of three control trials and seven test trials in randomised order (with control trials interspersed across session). There were four conditions: test step 1 quality, test step 2 quality, test step 1 quantity and test step 2 quantity. Half the subjects received step 1 before step 2, the other half received step 2 before step 1. Crow subjects were required to select correctly in 16 of 21 test trials to pass each condition. Each bird received up to three test sessions per day, depending on time constraints and motivation.

For the children, we ran 12 trials: 6 per condition, with 3 test trials and 3 control trials per condition, and 8 trials in set 1 and 4 trials in set 2. Both Experiments 1 and 2 of this study were run within the same session, amounting to 28–30 trials, lasting around 20 min. For the crows, all subjects were tested on both conditions (quality/quantity) in Experiment 1. However, if they failed both Experiment 1 conditions by selecting correctly in fewer than 16 out of 21 test trials in each condition (as this would be non-significant with a two-tailed exact Binomial test), they were discounted from Experiment 2. This approach was chosen because birds that failed the test when both rewards were visible (Exp 1) were extremely unlikely to pass when one or both rewards were not visible (Exp 2). If they failed one condition (e.g. quantity) but passed the other (e.g. quality) in Experiment 1, then they were tested in Experiment 2 only in the condition that they had previously passed (so quality in this case). For the children, all subjects were tested on Experiment 1 and Experiment 2 for both conditions, regardless of individual performance.

### Data analysis

We recorded the choice per trial for each subject as ‘correct’ or ‘incorrect’, with the correct choice being the reward of higher quality or quantity, whether it was immediate (control trial) or delayed (test trial). All test sessions were coded live as well as being video-recorded (unless parental consent requested otherwise for the children). 10% of trials were coded from video and compared to the live coding, finding 97.4% agreement with the human data, and 100% agreement for the crow data. Example trials can be found in the Online Resources.

We conducted Generalized Linear Mixed Models (GLMM: (Baayen 2008) using R (version 2.15.0; R Core Team 2014) to assess which factors influenced success rate in the New Caledonian crows and children. Success was a binary variable indicating whether the subject correctly solved the trial (1) or not (0) and was entered as a dependent variable in the models. For each species model in Experiment 1, we included the random effect of subject ID, fixed effects of age in years (children: continuous: ages 3–5 in individual years, crows: adult/juvenile), trial type (control, test trial), condition (quality, quantity), order (quality–quantity, quantity–quality), and gender (male/female). For Experiment 2, for the GLMM for the child data, we included the same fixed effects as Experiment 1 as well as adding the fixed effects of trial number (1–12) and visibility (immediate reward visible, delayed reward visible, neither reward visible). For the crows, we included the fixed effects of condition, trial type, order and visibility (immediate or delayed reward visible). We used likelihood ratio tests to compare the full model (all predictor variables, random effects and control variables) first with a null model, and then with reduced models to test each of the effects of interest (Forstmeier and Schielzeth 2011). The null model consisted of random effects, control variables and no predictor variables. The reduced model comprised of all effects present in the full model, except the effect of interest (Göckeritz et al. 2014). For the crows and children, we also analysed the data for the significant variables identified in the GLMMs using non-parametric two-tailed statistics, namely 1-sample Wilcoxon signed ranks tests and exact two-tailed Binomial tests run in SPSS version 21.

## Results

### Preference test

All crows completed both quality and quantity preference tests within 20 trials. In the quality preference test, eight subjects scored 20/20 and one subject 18/20, showing a clear preference for meat over apple rewards. In the quantity preference test, two subjects scored 18/20, four subjects 19/20 and two subjects 20/20, showing a preference for the larger over the smaller reward.

### Experiment 1: the influence of reward type

**Fig. 3:**
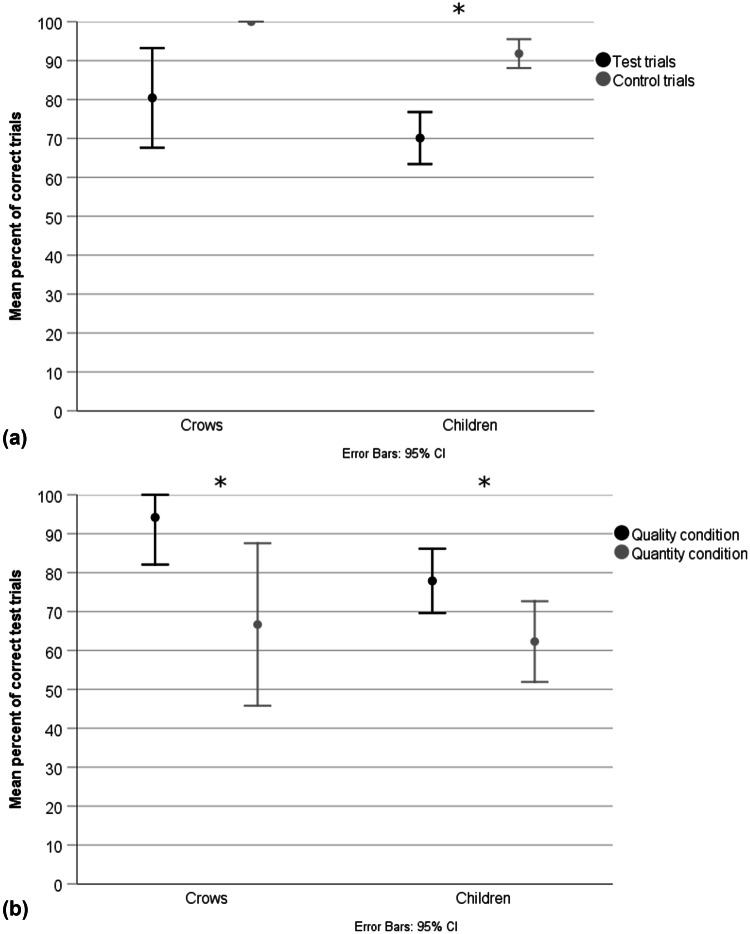
Experiment 1: **a** performance of crows and children in test and control trials; **b** test performance of crows and children in the quality and quantity condition. Performance above or below chance is shown, whereby the chance to choose correctly in this two-choice design is 50%. * indicates significant differences in performance across trial type and condition

All nine birds passed the training for Experiment 1 and were, therefore, used in the test. In the test and control trials, the full models differed significantly from the null models (crows: *χ*^2^ = 59.83, *df* = 4, *p *= < 0.001; children: *χ*^2^ = 49.989, *df* = 4, *p *= < 0.001). For the crows, we found a significant main effect of condition (quality vs. quantity) and order (quality–quantity vs. quantity–quality; Table 1). There was no significant effect of trial type (test vs. control), however, all crows picked the first, more preferred reward over the delayed, less preferred reward in all control trials in Experiment 1. For the children, we found a significant main effect of condition and trial type (test vs. control; Table 1). Specifically, children performed better in the control than test trials (Fig. 3a). Crows and children performed better in the quality than quantity condition (Fig. 3b). The crows and children performed significantly above chance within each condition (1-sample Wilcoxon signed ranks test: crows: quality: *p* = 0.005; quantity: *p* = 0.017; children: quality: *p* = < 0.001; quantity: *p* = < 0.001). Additionally, the birds in the subgroup tested in order quality–quantity performed better than those in subgroup with order quantity–quality.

**Table 1:** Experiment 1: generalized linear mixed models on factors affecting the number of correct test and control trials in New Caledonian crows (*n* = 9) and children (*n* = 61)

Fixed term	New Caledonian crows			Children		
	Estimate	*z* value	*p* value	Estimate	*z* value	*p* value
Trial type	19.219	0.033	0.974	1.756	5.953	**< 0.001**
Condition	− 2.105	0.349	**< 0.001**	− 0.561	− 2.152	**0.031**
Age in years	− 0.936	0.45	0.05	0.342	1.537	0.124
Order	1.219	0.445	**0.006**	− 0.040	− 0.115	0.910
Gender	0.451	0.478	0.345	0.223	0.623	0.534

Significant *p* values are highlighted in bold

### Experiment 2: the influence of reward visibility

**Fig. 4:**
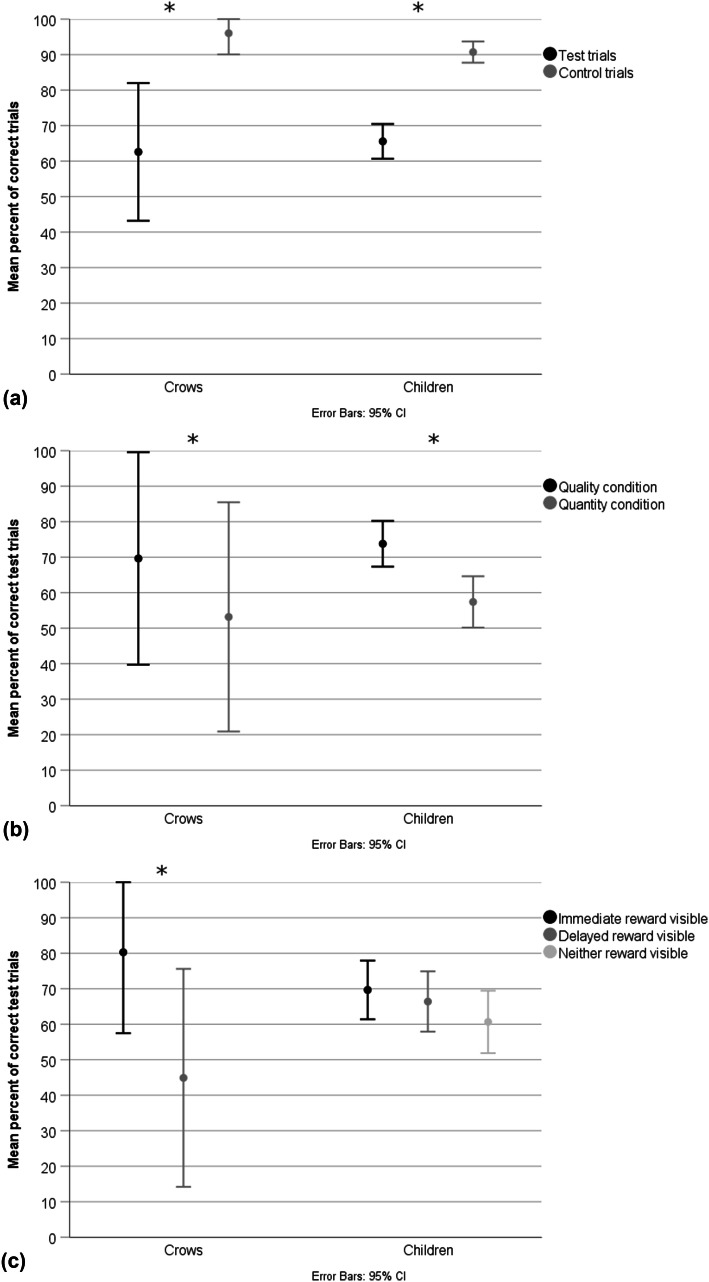
Experiment 2: **a** performance of crows and children in test and control trials; **b** test performance of crows and children in the quality and quantity condition; **c** test performance of crows and children depending on reward visibility. Performance above or below chance is shown, whereby the chance to choose correctly in this two-choice design is 50%. * indicates significant differences in performance

Four of the nine birds failed the training for Experiment 2. Therefore, five birds were tested. In the test and control trials, the full models differed significantly from the null models (crows: *χ*^2^ = 62.32, *df* = 3, *p* = < 0.001; children: *χ*^2^ = 94.48, *df* = 4, *p* = < 0.001). For the crows, we found a significant main effect of trial type (test vs. control), condition (quality vs. quantity), order (quality–quantity vs. quantity–quality) and visibility (immediate vs. delayed reward visible; Table 2). For the children, we found a significant main effect of trial type, condition and age (3–5 years; Table 3). Specifically, the crows and children performed better in the quality over quantity condition (Fig. 4b), and in the control over test trials (Fig. 4a). 5-year-old children performed significantly better than 3- and 4-year olds.

**Table 2:** Experiment 2: generalized linear mixed models on factors affecting the number of correct test and control trials in crows

Fixed term	Estimate	*z* value	*p* value
Trial type	3.55	6.212	**< 0.001**
Condition	− 0.775	− 2.469	**0.014**
Order	− 1.755	− 4.011	**< 0.001**
Visibility	− 1.697	− 5.803	**< 0.001**

*N* = 5

Significant *p* values are highlighted in bold

**Table 3:** Experiment 2: generalized linear mixed models on factors affecting the number of correct test and control trials in children

Fixed term	Estimate	*z* value	*p* value
Trial type	2.331	5.213	**< 0.001**
Condition	− 0.684	− 3.308	**0.001**
Age in years	0.432	2.182	**0.029**
Order	0.187	0.603	0.546
Gender	− 0.148	− 0.463	0.643
Trial number	− 0.156	− 1.256	0.209
Visibility	0.267	0.428	0.668

*N* = 61

Significant *p* values are highlighted in bold

With regard to reward visibility, the crows performed better when only the immediate reward was visible over when only the delayed reward was visible (Fig. 4c). Specifically, on the group level, the crows performed significantly above chance when only the immediate reward was visible (delayed reward not visible), but not when the delayed reward was visible (immediate reward not visible), and did not perform significantly above chance within either condition (quality/quantity; Table 4). Reward visibility had no significant effect on the children’s performance (Table 3; Fig. 4). Children of each age performed significantly above chance across both conditions, and within each condition across age groups (Table 5). Additionally, crows also performed better with the starting order quality*–*quantity over quantity*–*quality. The crow training performance is reported in Online Resource 1.

**Table 4:** Experiment 2: performance of the crows

Visibility/condition	Z	*p*
Immediate reward visible (*n* = 7)	2.226	**0.026**
Delayed reward visible (*n* = 7)	1.185	0.236
Quality condition (*n* = 4)	1.841	*0.066*
Quantity condition (*n* = 3)	1.069	0.285

Results reflect 1-sample Wilcoxon signed ranks tests, with *n* signifying the number of tests

Significant results highlighted in bold

**Table 5:** Experiment 2: performance for children within age groups and conditions

Age/condition	Z	*p*
Age 3 (*n* = 20)	3.73	**< 0.001**
Age 4 (*n* = 21)	3.535	**< 0.001**
Age 5 (*n* = 20)	3.848	**< 0.001**
Quality condition (*n* = 61)	6.402	**< 0.001**
Quantity condition (*n* = 61)	5.624	**< 0.001**

Results reflect 1-sample Wilcoxon signed ranks tests, with *n* signifying the number of tests

Significant results highlighted in bold

### Crow individual-level performance

We focussed primarily on group-level analyses for the humans and crows; however, we present the individual-level analyses for the crows (Table 6). In Experiment 1, eight of nine crows passed the quality condition and seven of nine crows passed the quantity condition. In Experiment 2, for quality condition, four of four subjects tested passed step 1 when the immediate reward was visible, and one of four passed step 2 when the delayed reward was visible. For the quantity condition, two of three subjects tested passed step 1, and one of three passed step 2.

**Table 6:** Performance in test trials and control trials for the crows

Subject	Exp 1 quality	Exp 1 quantity	Exp 2 quality step 1	Exp 2 quality step 2	Exp 2 quantity step 1	Exp 2 quantity step 2
Jupiter	**< 0.001**	**< 0.001**	**–**	**–**	**–**	**–**
Mars	0.099	**< 0.001**	**–**	**–**	1.000	0.200
Triton	**< 0.001**	**0.001**	**< 0.001**	0.200	**< 0.001**	0.362
Neptune	**< 0.001**	**< 0.001**	**< 0.001**	**< 0.001**	**0.016**	**< 0.001**
Io	**< 0.001**	**0.016**	**–**	**–**	**–**	**–**
Mercury	**< 0.001**	0.585	**< 0.001**	*0.043*	**–**	**–**
Venus	**< 0.001**	**0.016**	**< 0.001**	0.362	**–**	**–**
Uranus	**< 0.001**	1.000	**–**	**–**	**–**	**–**
Saturn	**< 0.001**	**< 0.001**	**–**	**–**	**–**	**–**

Results reflect binomial exact two-tailed tests

Bold = significant preference for the correct choice (delayed most preferred reward); italics = significant preference for the incorrect choice (immediate less preferred reward)

### Behavioural strategies to aid delayed gratification

We observed both species using strategies to track rewards on the rotating tray in both experiments. Some children tracked preferred rewards by pointing a finger at them while the tray was rotating, and verbally confirming which reward they preferred. Crows often jumped onto the table next to the apparatus while appearing to visually track the delayed reward or perched on a branch further away from the apparatus until the preferred reward was in reach. We did not observe any other distraction techniques.

## Discussion

We investigated delayed gratification as a measure of self-control in corvids and children using an adaptation of the rotating tray paradigm introduced by Bramlett et al. (2012). The authors acknowledge that confounds exist in the study design, such as differences between the two species in training, reward types and observation of apparatus baiting in Experiment 2. Therefore, species cannot be isolated as the cause of group differences, as any of these confounds likely affected performance. Due to these differences in methodology, we cannot compare species directly, but describe both species’ performances in both experiments.

In Experiment 1, we found that, similarly with most capuchin monkeys tested in this previous study (Bramlett et al. 2012; Perdue et al. 2015), both New Caledonian crows and children were able to inhibit taking an immediate, less preferred reward to wait for a delayed (15 s), more preferred option. With regard to reward type, on the group level, both species were more likely to delay gratification when rewards differed in quality than in quantity, though both species performed above chance in both conditions. In Experiment 2, when reward visibility was manipulated, the ability to wait for the delayed reward depended on the types of rewards presented. Both species performed better when rewards differed in quality over quantity, and children, though not crows, performed above chance in both conditions. With regard to reward visibility, crows performed better when the immediate reward was visible than when the delayed reward was visible. Children showed no difference in performance depending on reward visibility, which is similar to a previous study with capuchin monkeys (Perdue et al. 2015), though the children did show an age effect on performance in Experiment 2 as 5-year olds outperformed 3- and 4-year olds. Although we focussed primarily on group-level analyses, we found that on the individual level, most crows performed similarly well in preference testing and Experiment 1, but most individuals struggled with Experiment 2 where reward visibility was manipulated, likely due to them not observing the baiting of the apparatus and thus relying in inference to make choices during the test.

Previous studies in corvids, parrots and chimpanzees indicate that while these species succeed in tasks that require delaying gratification to gain higher quality rewards, the subjects struggle when the delayed reward is of higher quantity (Auersperg et al. 2013; Beran et al. 2016b; De Petrillo et al. 2015; Dufour et al. 2007, 2012; Hillemann et al. 2014), though few studies have compared between taxa using similar paradigms. Our results confirm this pattern in New Caledonian crows and 3–5-year-old children, who were more likely to wait for better quality over higher quantity rewards in both experiments. When given a choice between different quantities of the same food, corvids may select the smaller amount, as it is easier to eat, carry and cache nearby, which may contribute to poorer performance in quantity-based delayed gratification tasks in these species. Jungle crows (*Corvus macrorhynchos*) can reliably select a larger over smaller quantity (Bogale et al. 2011) and the New Caledonian crows in our study were able to do so during the preference testing (1 vs. 4 choice). However, carrion crows did not choose the larger quantity significantly from chance when presented with 1 vs. 2 choice, though did so in 1 vs. 4 choice (Bugnyar et al. 2012) and Eurasian jays (*Garrulus glandarius*) only reliably chose the larger option in a 1 vs. 6 choice (unpublished data). Our results, therefore, suggest that the actual choices presented during delayed gratification testing may influence performance in both corvids and children. In both experiments, crows that started with the quality condition performed significantly better than crows that started with the quantity condition, while no such difference was found for children. Crows also performed better in the quality condition than the quantity condition in both experiments. Thus, receiving the ‘easier’ condition (quality) before the ‘harder’ condition (quantity) may have helped their overall performance by giving them more experience with the paradigm and of successfully obtaining the ‘better’ reward, while this was not the case for children. Task difficulty can affect a subject’s expectations and perceived ability in further tasks (Kumar and Jagacinski 2011). Therefore, higher success in the quality condition when it was presented first may have enhanced the crows’ performance in the quantity condition by altering their expectations.

In Experiment 1 (reward type manipulated), both species performed significantly above chance for the quantity and quality conditions. This suggests that the ability to let one reward pass to get another is also evident in more distantly related species to humans, indicating that we need to expand the phylogenetic assessment of delayed gratification and choice behaviour using this task, as well as variations of this task, to explore where species differ or perform similarly. However, only children performed significantly above chance for all conditions in Experiment 2 (reward visibility also manipulated), while the crows’ performance was dependent on the visibility of the rewards. In humans and great apes, delay of gratification is typically easier when the delayed, most preferred reward is visible, rather than the immediate, less preferred reward (Kochanska et al. 2001; Vlamings et al. 2006). This is likely due to being able to accurately predict and focus attention on the delayed reward when it is visible. In contrast, in Perdue et al. (2015) using the rotating tray paradigm, capuchin monkeys showed no difference in performance whether the first or second reward was visible. The authors highlight that, unlike most other paradigms, temporal delays are intuitive in this paradigm as the subject is able to directly observe the rewards moving closer to them when both rewards are visible as in Experiment 1 of this study (Perdue et al. 2015). In our study, New Caledonian crows performed better when the immediate reward was visible over when only the delayed reward was visible. This suggests that the birds may make a decision when they could see the first reward without also having to pay attention to the second reward. Therefore, if the reward under the first, clear lid was visible, they could decide to wait for the second reward at that point, while if the first location was hidden, they had to attend to the second location from the start. This was also apparent during training, where several individuals always chose the first lid if it was opaque, i.e. not paying attention to the second, clear lid, but made the correct choices, i.e. taking it or leaving it to wait for the second, opaque lid, if the first lid was clear.

Children, on the other hand, showed no difference in performance when reward visibility was manipulated—which is similar to that of the capuchin monkeys in a previous study with this paradigm (Perdue et al. 2015). This may in part be due to the children observing the experimenters’ hands during baiting, and thus remembering which reward was placed in which location. This was confirmed in test type 3 of Experiment 2 when both rewards were hidden. Studies on New Caledonian crows indicate that they do not track human hand movements without specific training (Jelbert et al. 2016). Although the birds were always given the opportunity to observe baiting, most did so from a perch above the table instead of sitting directly at the table while the experimenter was present and baiting and approached the apparatus only when the experimenter had left the aviary. Hence, the crows had to take into account which reward type was visible and then potentially to rely on inference by exclusion to locate the more preferred reward. When baiting visibility was removed, capuchin monkeys were still able to wait for the larger, delayed reward in the rotating tray task when it was not visible (Perdue et al. 2015). Similarly, in a previous study, New Caledonian crows were able to make inferences by exclusion to find hidden food in a choice task (Jelbert et al. 2015). Furthermore, most, but not all, crows were able to pass the Experiment 2 training, where they had to successfully locate one reward hidden under a transparent or opaque container. It is possible that this testing context, where the crows were also required to make decisions about taking an immediate or delayed reward varying in quality or quantity, as well as either hand tracking and/or reasoning by exclusion resulted in a mental overload of working memory capacity and/or attentional allocation. Future studies may include some extensive pre-testing and hand tracking training for the crows to explore this possibility and to test the role of baiting visibility for the children. For example, crows could be trained to observe baiting similar to a study by Jelbert and colleagues (Jelbert et al. 2016), and children could be prevented from observing baiting, thus investigating the influence of observation, memory and ability to infer where each reward was placed.

Both children and crows showed behavioural strategies to track rewards on the rotating tray, such as pointing a finger at the preferred reward or sitting on the table next to the apparatus to visually track the reward. While distraction can be a useful strategy to improve self-control and has been shown in many species such as chimpanzees (Evans and Beran 2007), children (Steelandt et al. 2012), carrion crows, common ravens (Dufour et al. 2012) and kea (Schwing et al. 2017), we did not see evidence of similar types of distraction techniques in our study, which is likely due to the nature of the paradigm as, unlike some other paradigms like the exchange paradigm, the subjects did not have access to the initial reward. Additionally, there was only a short delay in comparison to other paradigms, though this delay could be extended in future work by slowing down the tray speed and changing the reward positioning.

Previous studies have shown that executive function, including self-control, in humans improves between ages 3–5 years with significant improvement by 4-year old and above (Carlson et al. 2005; Hughes 1998; Kochanska et al. 2001; Zelazo 2003), although children differentiate between rewards of different values from 1-year old (Butterworth 2005). While there was no age effect for children in Experiment 1, 5-year-old children outperformed 3- and 4-year olds in Experiment 2. Delays in this study were short compared to previous work with children (Steelandt et al. 2012); therefore, even children as young as 3-years old were able to wait for the delayed reward in Experiment 1. We selected a short delay of 15 s in the present study, as we replicated the methodology used with capuchin monkeys previously tested in this paradigm (Bramlett et al. 2012; Perdue et al. 2015)—future studies could increase the delay to investigate its influence on performance. The difference in performance between age groups is likely due to reward visibility, as in Experiment 1 both rewards were visible at the same time, while in Experiment 2 either one or both rewards were hidden. Thus, the children could use memory and inference to determine which location on the rotating tray contained the preferred reward. Reward visibility also negatively impacted on performance in New Caledonian crows, who did not observe baiting and, therefore, did not use memory, but rather inference alone to make their choices. Future studies may investigate the effect of memory and inference on success rates in similar tasks.

Few studies have explored whether delayed gratification is consistent within an individual when tested using different paradigms. In capuchins, individuals tested on the rotating tray and accumulation paradigm were more proficient on the rotating tray than accumulation task (Evans et al. 2012), and also showed improved self-control overall likely due to greater experience of delayed gratification tasks (Beran et al. 2016a). Future studies could similarly compare delay of gratification paradigms, including the rotating tray with other paradigms, with one another within the same sample and species, which, to our knowledge, has yet to be done with most species, including New Caledonian crows and children. This approach would enable researchers to validate and compare these different tasks and indicate whether the ability to delay gratification is consistent across tasks for the same individuals or differs depending on other aspects such as age or experience. Familiarity and apparent trustworthiness of the experimenter may influence choices and understanding of time—from around age 4 or 5 when children can differentiate future and past events (Atance and O’Neill 2001). In humans, the social component in exchange tasks may contribute to waiting tolerance, as children are encouraged to be patient and prosocial, such as waiting until the end of a meal for dessert and sharing toys with siblings (Steelandt et al. 2012). As such, it would be of interest in future studies, to compare individual performance in task that do not have social components, like the present rotating tray one, with those that do, like exchange tasks.

## Conclusion

In conclusion, we present the first study to explore delay of gratification as a measure of self-control in New Caledonian crows and 3–5-year-old children, while manipulating reward type and reward visibility using a novel rotating tray paradigm. We describe both species’ performance using the rotating tray paradigm, though we note differences in methodology for each species, such as training and reward types, which are detailed in the “Materials and methods” section. In Experiment 1, both species were able to show self-control when both rewards were visible—i.e., the children and the corvids could refrain from selecting the immediate reward for the delayed one when the delayed reward was of higher value. This supports findings from previous studies with other paradigms, like the exchange paradigm, where children and other corvid species were tested in separate studies (Dufour et al. 2012; Steelandt et al. 2012). In both experiments, children and corvids performed better when rewards differed in quality over quantity, though did select correctly above chance in both conditions. Children had only previously been tested using rewards of varying quantity—and not in direct comparisons to rewards of varying quality.

In Experiment 2, the crows and children performance differed when reward visibility was manipulated. Specifically, the crows’ performance was influenced by reward visibility while the children’s performance was not, indicating that children may have used observation and memory to determine the location of the preferred reward, while crows needed to make decisions by inference based on the visible reward. The crow performance was likely due to crows not observing human hand movements without extensive training (Jelbert et al. 2016), and thus not paying attention to the baiting of the apparatus in this study and relying on inference alone. Crows performed better when tested in the quality condition first and quantity condition second, while there was no difference for children, indicating an effect of experience for the crows but not for children, which is likely due to differences in pre-test experience. These results contribute to our understanding of self-control in birds and humans, and particularly, to some of the contextual factors that may influence performance in these tasks. These factors should be taken into account when designing future experiments and when comparing performance between different species. Hence, our findings help to provide a base for future research into the mechanisms of self-control.

## Electronic supplementary material

Below is the link to the electronic supplementary material.

Supplementary material 1 (DOCX 22 kb)

Supplementary material 2 (MP4 39146 kb)
