# Supplementary material for: Delayed gratification in New Caledonian crows and young children: influence of reward type and visibility
Source: Anim Cogn. 2019 Oct 19;23(1):71–85. doi: 10.1007/s10071-019-01317-7 (PMC6981108; doi:10.1007/s10071-019-01317-7)
Supplement: Supplementary file 1 — Supplementary material 1 (DOCX 22 kb) [file 10071_2019_1317_MOESM1_ESM.docx]

**ONLINE RESOURCES**

**Delayed gratification in New Caledonian crows and young children: influence of reward** **type and visibility**

Rachael Miller ^1^, Anna Frohnwieser ^1^, Martina Schiestl ^2,3^, Dakota E. McCoy^4^, Russell D. Gray ^2,3^, Alex H. Taylor ^2^, Nicky S. Clayton ^1^

^1^ Department of Psychology, University of Cambridge, Cambridge, UK

^2^ School of Psychology, Auckland University, Auckland, New Zealand

^3^ Max Planck Institute for the Science of Human History, Max Planck Society, Jena, Germany

^4^ Department of Organismic and Evolutionary Biology, Harvard University, Cambridge, USA

Online Resource 1. Crow subject information and testing order. M = male, F = female

| **ID** | **Sex** | **Age** | **First condition tested** |
| --- | --- | --- | --- |
| Jupiter | M | Juvenile | Quantity |
| Mars | M | Juvenile | Quantity |
| Tritone | F | Juvenile | Quality |
| Neptune | F | Juvenile | Quality |
| Io | M | Adult | Quality |
| Mercury | F | Adult | Quantity |
| Venus | M | Juvenile | Quality |
| Uranus | F | Adult | Quantity |
| Saturn | M | Adult | Quality |

Online Resource 2. Training performance of crows. Experiment 1: All subjects passed the training. Experiment 2: 7 of 9 subjects passed training 1 (both locations visible) training and 5 of these 7 subjects passed training 2 (only one location visible) training. As one of these 5 subjects failed the quality condition test in Experiment 1, 4 subjects proceeded to testing in the quality condition in Experiment 2. As two of these 5 subjects failed the quantity condition test in Experiment 1, 3 subjects proceeded to testing in the quantity condition in Experiment 2.

| **Subject** | **No. of sessions to pass Experiment 1 training** | **No. of sessions to pass Experiment 2 training 1** | **No. of sessions to pass Experiment 2 training 2** | **Tested in Experiment 2** |
| --- | --- | --- | --- | --- |
| Jupiter | 2 | 4 | failed | No - Failed training |
| Mars | 2 | 3 | 10 | Yes - Quantity |
| Triton | 2 | 3 | 2 | Yes - Quality, quantity |
| Neptune | 2 | 2 | 6 | Yes - Quality, quantity |
| Io | 3 | failed | - | No - Failed training |
| Mercury | 2 | 4 | 2 | Yes - Quality |
| Venus | 2 | 5 | 8 | Yes - Quality |
| Uranus | 5 | 5 | failed | No - Failed training |
| Saturn | 3 | failed | - | No - Failed training |
